# Exogenous glycogen utilization effects the transcriptome and pathogenicity of *Streptococcus suis* serotype 2

**DOI:** 10.3389/fcimb.2022.938286

**Published:** 2022-11-09

**Authors:** Mei-Fang Tan, Jia Tan, Fan-Fan Zhang, Hai-Qin Li, Hua-Yuan Ji, Shao-Pei Fang, Cheng-Cheng Wu, Yu-Ling Rao, Yan-Bin Zeng, Qun Yang

**Affiliations:** Institute of Animal Husbandry and Veterinary Science, Jiangxi Academy of Agricultural Sciences, Nanchang, China

**Keywords:** *Streptococcus suis*, exogenous glycogen, ApuA, pathogenicity, virulence factor, carbon metabolism

## Abstract

*Streptococcus suis* serotype 2 (SS2) is an important zoonotic pathogen that causes severe infections in humans and the swine industry. Acquisition and utilization of available carbon sources from challenging host environments is necessary for bacterial pathogens to ensure growth and proliferation. Glycogen is abundant in mammalian body and may support the growth of SS2 during infection in hosts. However, limited information is known about the mechanism between the glycogen utilization and host adaptation of SS2. Here, the pleiotropic effects of exogenous glycogen on SS2 were investigated through transcriptome sequencing. Analysis of transcriptome data showed that the main basic metabolic pathways, especially the core carbon metabolism pathways and virulence-associated factors, of SS2 responded actively to glycogen induction. Glycogen induction led to the perturbation of the glycolysis pathway and citrate cycle, but promoted the pentose phosphate pathway and carbohydrate transport systems. Extracellular glycogen utilization also promoted the mixed-acid fermentation in SS2 rather than homolactic fermentation. Subsequently, *apuA*, a gene encoding the unique bifunctional amylopullulanase for glycogen degradation, was deleted from the wild type and generated the mutant strain Δ*apuA*. The pathogenicity details of the wild type and Δ*apuA* cultured in glucose and glycogen were investigated and compared. Results revealed that the capsule synthesis or bacterial morphology were not affected by glycogen incubation or *apuA* deletion. However, extracellular glycogen utilization significantly enhanced the hemolytic activity, adhesion and invasion ability, and lethality of SS2. The deletion of *apuA* also impaired the pathogenicity of bacteria cultured in glucose, indicating that ApuA is indeed an important virulence factor. Our results revealed that exogenous glycogen utilization extensively influenced the expression profile of the *S. suis* genome. Based on the transcriptome response, exogenous glycogen utilization promoted the carbon adaption and pathogenicity of SS2.

## Introduction

*Streptococcus suis* is one of the most important bacterial pathogens responsible for significant economic losses in the global swine industry. As an opportunistic pathogen, *S. suis* asymptomatically colonizes the mucosal surfaces of the oropharynx, gastrointestinal tract, or respiratory tract of almost all pigs (Gottschalk et al., 2010). *S. suis* causes a wide variety of severe clinically features, mainly including meningitis, septicemia, arthritis, and endocarditis (Feng et al., 2014). *S. suis* is also an emerging zoonotic agent that has caused three deadly outbreaks in humans and has claimed at least 1,600 human lives within the last decade (Dong et al., 2021). Among the 33 serotypes originally classified based on the antigenicity of capsular polysaccharide (CPS), *S. suis* serotype 2 (SS2) has gained more attention because of its high virulence and prevalence worldwide (Doran et al., 2016).

Carbohydrate adaption and availability in different host niches is an essential step for free-living pathogenic bacteria to ensure the survival and growth, such as *Escherichia coli* (Tian et al., 2013), *Streptococcus mutans* (Klein et al., 2010; Moye et al., 2014), *Streptococcus pneumoniae* (Abbott et al., 2010; Marion et al., 2011), and *S. suis* (Wu et al., 2014; Willenborg et al., 2015), during the establishment of invasive infection. Accordingly, pathogens possess efficient stress-response mechanisms to adapt to various conditions and ensure their growth and proliferation (Zhang et al., 2016). The selective carbon-source utilization in bacteria is common, and many bacteria preferentially use glucose, which is easily accessible and allows the fastest growth (Gorke and Stulke, 2008). This utilization of preferred carbon sources over other carbon sources is tightly regulated in a hierarchical order by carbon catabolite repression (CCR) and carbon catabolite activation (CCA). Catabolite control protein A (CcpA) is a transcriptional mediator of CCR and CCA in Gram-positive bacteria. CcpA is an important regulator of carbohydrate-dependent metabolic switches and the regulator for a balanced metabolic flux in the central carbon metabolism (Willenborg et al., 2014).

Metabolic adaptation is closely related to the virulence of pathogenic bacteria. Elements of CCR are crucial to the expression of virulence genes and thus to pathogenicity (Gorke and Stulke, 2008). Proteins that are involved in the use of alternative nutrients are often virulence factors, such as CcpA (Willenborg et al., 2011). Moreover, different exogenous carbohydrates induce different transcriptional and metabolic effects on pathogens (Paixao et al., 2015). Pullulan polysaccharides reportedly affect the metabolic pathways and virulence gene expression of SS2, raising the possibility that starch in animal feeds could influence the switch from an asymptomatic to a pathogenic association of *S. suis* when colonized in porcine oropharynx (Ferrando et al., 2014).

In mammals, glycogen primarily exists in the liver and skeletal muscle. Other tissues such as myocardium and kidney also contain a small amount of glycogen. Glycogen is also found in the mammalian brain and accounts for about 0.1% of the total brain weight (Brown, 2004). Glycogen is distributed uniformly throughout the brain and stored primarily in grey-matter structures (Brown, 2004). Results of a CD1 mouse infection model have proposed the abundance of glycogen in areas where SS2 invades and colonizes the host brain (Dominguez-Punaro et al., 2007), suggesting the possible spatial associations between glycogen distribution and *S. suis* infection. Otherwise, the glycogen released from damaged cells may support the growth of *S. suis* in host organs and tissues (Ferrando et al., 2014). However, knowledge on the internal connection between the glycogen utilization and host adaptation of *S. suis* remains limited to date.

In this study, we investigated the role of exogenous glycogen utilization in the metabolic adaptation and virulence properties of the SS2 strain. We performed transcriptome comparative analysis for *S. suis* growth on glucose or glycogen and identified differentially expressed genes (DEGs), especially the core carbon metabolism components and virulence-associated factors. ApuA is a cell wall-anchored protein and is a unique bifunctional amylopullulanas that plays a critical role in the degradation of α-glucans in *S. suis* (Ferrando et al., 2010). Thus, we constructed an *apuA*-deletion mutant (Δ*apuA*) and compared the virulent phenotypes between the wild-type (WT) and Δ*apuA* in the presence of glucose and glycogen, respectively. The analyses of transcriptome data and pathogenic assays revealed that glycogen utilization affected the metabolic network of *S. suis* and positively regulated the virulence gene expression and pathogenicity of SS2.

## Materials and methods

### Bacterial strains, plasmids, and growth conditions

The bacterial strains and plasmids used in this study are listed in **Table 1**. WT is a highly virulent SS2 strain SC19 isolated from a sick pig during the 2005 epidemic outbreak in Sichuan, China (Tan et al., 2015). In general, SS2 strains were grown at 37°C in tryptone soya broth (TSB; Difco, Le Pont de Claix, France) or on tryptic soy agar (TSA; Difco) with 5% (*v*/*v*) fetal bovine serum (Sijiqing, Hangzhou, China). *E*. *coli* strain DH5α was grown at 37°C in lysogeny broth (Difco). A complex medium (Ferrando et al., 2010) was used to assess growth on different carbohydrate sources by supplementation with glucose or glycogen at a final concentration of 1% (*w*/*v*). The complex medium without additional carbon sources was prepared as negative control during growth assays. Growth in different media was determined by measurement of turbidity at an optical density of 600 nm (OD_600_). When constructing the *apuA*-deletion mutant of SS2, erythromycin (50 μg/mL) and spectinomycin (100 μg/mL) were supplemented. Unless otherwise specified, carbohydrates, antibiotics, and chemicals were purchased from Sigma–Aldrich (Shanghai, China).

**Table 1:** Bacterial strains and plasmids used in this study.

Strains/plasmids	Characteristics*^a^*	Source
Bacterial strains		
SC19	Virulent Chinese *S. suis* serotype 2 isolate; wild-type (WT)	(Tan et al., 2015)
Δ*apuA*	SC19 *apuA*::*erm* (Erm^r^)	This study
*E. coli* DH5α	Cloning host for recombinant plasmids	Trans
Plasmids		
pAT18	The plasmid carrying an erythromycin resistance rRNA methylase (*erm*) gene expression cassette	(Trieu-Cuot et al., 1991)
pSET4s	Temperature-sensitive *E. coli*- *S. suis* shuttle vector (Spc^r^)	(Takamatsu et al., 2001)
pSET4s-apuA	Derived from pSET4s for knocking out *apuA* in SC19 (Spc^r^Erm^r^)	This study

**^a^** Erm^r^, erythromycin resistant; Spc^r^, spectinomycin resistant.

### Transcriptome sequencing and analysis

For RNA extraction, SC19 was cultured in a complex medium with 1% glucose and 1% glycogen to the mid-log phase (an OD_600_ of ~0.5). RNA was obtained using SV Total RNA Isolation System (Promega, Wisconsin, USA) according to the manufacturer’s instructions. The quality and concentration of RNA were monitored by Eppendorf BioPhotometer plus.

RNA sequencing was performed in Shanghai Personal Biotechnology Co.,Ltd by using Illumina HiSeq 2000. Quality control analysis of raw data was conducted using FastQC (http://www.bioinformatics.babraham.ac.uk/projects/fastqc). Clean reads were mapped onto the complete reference *S. suis* SC84 genome in NCBI (GenBank accession no. NC_012924.1) by using Bowtie2 (http://bowtie-bio.sourceforge.net/index.shtml). SC19 has similar background to *S. suis* SC84, which is a human-origin strain isolated in the 2005 outbreak of Sichuan China (Sun et al., 2008). DEseq (version 1.18.0) was used to analyze differences in gene expression (Anders and Huber, 2010). The main parameters were set as follows: log_2_|fold change| > 1 and *P*-value < 0.05. Enrichment analysis was performed based on the Kyoto Encyclopedia of Genes and Genomes database (KEGG, https://www.kegg.jp/) with the Blast2GO software (https://www.blast2go.org). The number of DEGs at different levels of each KEGG pathway was counted, and the main metabolic pathways and signaling pathways were determined with hypergeometric distribution method (Luo et al., 2009).

### Quantitative real-time PCR (qRT-PCR)

Twelve DEGs were selected to evaluate the reliability of transcriptome data through qRT-PCR detection. Primers are designed and listed in **Table S1**. Bacterial strain, culture conditions, and RNA extraction were carried out as described above. cDNA was synthesized using Reverse Transcription System (Promega). qRT-PCR assays were performed on an ABI prism 7900HT sequence detection system by using AceQ qPCR SYBR Green Master Mix (Vazyme, Nanjing, China) under the following parameters: 95°C for 5 min, followed by 40 cycles of 95°C for 15 s, and 60°C for 30 s. 16S rRNA served as the internal reference gene. Reactions were performed with three independent biological repeats. Relative expression level was measured with 2^−ΔΔCt^ method (Livak and Schmittgen, 2001). Data were presented as mean ± standard deviation between glucose and glycogen cultures.

### Construction and identification of mutant strain

Thermosensitive suicide vector pSET4s was used for gene replacement in SC19 according to the homologous recombination method as previously described (Takamatsu et al., 2001). All the primers are listed in **Table S1**. Primers Aup-F/Aup-R and Adown-F/Adown-R were used for cloning the upstream and downstream homologous regions of the promoter and the N-terminal of *apuA*, respectively. The erythromycin-resistance gene expression cassette was amplified from pAT18 with primers Erm-F/Erm-R. Three DNA fragments were cloned into pSET4s sequentially to achieve the recombinant shuttle vector pSET4s-*apuA*. pSET4s-*apuA* was electroporated into strain SC19 to acquire the isogenic mutant strain Δ*apuA*. The bacterial colony that showed resistance to erythromycin and sensitivity to spectinomycin was recognized as the candidate mutant strain through temperature screening.

The upstream gene *apuR*, the coding region of α-amylase of *apuA*, the coding region of pullulanase of *apuA*, and the downstream gene *sgaT* were amplified by PCR using the primer pairs apuR-F/apuR-R, amy-F/amy-R, pul-F/pul-R, and sgaT-F/sgaT-R, respectively, to confirm the mutant strain Δ*apuA* (**Table S1**). DNA sequencing was also performed to confirm the deletion of *apuA*.

The inactivation of the *apuA* gene was further verified at the transcriptional level. For RNA extraction, SC19 and Δ*apuA* were cultured in TSB to the log phase. RNA extraction and cDNA synthesis were carried out as described above. Four primer pairs used for DNA detection were also used for real-time PCR to identify whether the upstream and downstream genes of *apuA* are functioning normally.

### Measurement of NAD^+^ and NADH

SS2 strains were cultured to the mid-log phase in a complex medium containing 1% glucose and 1% glycogen. Bacteria were collected (1 × 10^6^ CFU per sample) and used to analyze the total intracellular amount of NAD**^+^,** NADH, and NAD**^+^**/NADH ratio. The assay was performed using a CheKine™ Micro Coenzyme I NAD(H) Assay Kit (Abbkine, Wuhan, China) according to the manufacturer’s instructions. The amount of NAD**^+^** and NADH was calculated according to the total protein concentration of each sample. Protein concentrations were measured using a Micro BCA protein assay kit (Cwbiotech, Beijing, China).

### Electron microscopy

SS2 strains were cultured in a complex medium containing 1% glucose and 1% glycogen. Cells were harvested at the mid-log phase and fixed with 2.5% glutaraldehyde at 4°C overnight. Scanning electron microscopy (SEM) and transmission electron microscopy (TEM) analyses were performed in accordance with previously described methods (Tan et al., 2017). SEM observation was performed by using a JSM-6390LV SEM (NTC, Tokyo, Japan). TEM observation was performed by using an H-7650 TEM (Hitachi, Tokyo, Japan). Forty bacterial cells from each strain and each growth condition were randomly chosen from the TEM images to measure cell length, cell width, cell wall thickness, and capsule thickness by using the automated MicrobeTracker software (version 0.937).

### Titration of hemolytic activity

Hemolytic activity was detected in accordance with previously described methods (Ferrando et al., 2014). SS2 strains were grown in a complex medium containing 1% glucose and 1% glycogen. The supernatant was collected from each culture every 1 h through centrifugation at 12,000 ×g for 1 min. Defibrinated sheep blood (HyClone, Utah, USA) was centrifuged, washed, and resuspended in phosphate-buffered saline buffer to a final density of 2% (*v*/*v*). Each 100 μL of the supernatant was incubated for 2 h at 37°C with 100 μL of 2% sheep erythrocytes. Analyzed erythrocytes were precipitated by centrifugation at 1500 g for 15 min. About 100 μL of the supernatant was then transferred to a sterile new microplate and measured at 550 nm with a microELISA reader (Biotek, Vermont, USA). The absorbance values of blank glycogen and glucose were different, thus sterile culture media were used as negative controls and their absorbance values were deducted from that of the actual experimental data. The emendated experimental data were subsequently compared and analyzed.

### Adhesion and invasion assays

Adherence and invasion assays were conducted in accordance with previously described methods (Ferrando et al., 2010; Ferrando et al., 2014). SS2 strains were cultured in glucose and glycogen to the mid-log phase. Newborn pig tracheal cells (NPTr) were maintained in Dulbecco’s modified Eagle medium with high glucose (HyClone) supplemented with 10% fetal bovine serum (*v*/*v*) (HyClone) at 37°C and 5% CO_2_. For adherence assay, bacteria were added to the cell culture at a multiplicity of infection of ~ 100 and incubated at 37°C for 2 h. For the invasion assay, NPTr cells were incubated with ampicillin (100 μg/mL) for 2 h before lysis.

### Biofilm formation assay

Biofilm formation assay was conducted according to previously reported methods with few modifications (Tan et al., 2017). The overnight cultures of SS2 strains in glucose, glycogen, and complex medium were diluted 100-fold with fresh medium and added to a sterile 96-well microplate. Bacteria were incubated at 37°C for 24 h. The medium and free-floating bacteria were removed. After fixation by formaldehyde for 15 min, 100 μL of 0.04% crystal violet was added to each well and stained for 10 min. The wells were washed to remove the unbound crystal violet and dried at 37°C for 10 min. Each well was then added with 95% ethanol and shaken for 10 min to dissolve crystal violet. Absorbance was recorded at 550 nm with the microELISA reader. The wells with a sterile medium and complex medium were used as negative controls.

### Pathogenicity test

The *Galleria mellonella* (wax worm) model is simple and cost-efficient and allows experiments to be performed at the host temperature (Cutuli et al., 2019). The use of the *G. mellonella* infection model can effectively reduce the use of vertebrates. *G. mellonella* has been investigated for modeling a number of veterinary microbial pathogens, such as *E. coli* and *S. aureus* (Cutuli et al., 2019), group A streptococcus (Loh et al., 2013), and *S. suis* (Velikova et al., 2016). Therefore, the *G. mellonella* infection model was used in this study to detect the role of extracellular glycogen metabolism and ApuA in *S. suis* virulence.

The experimental procedure was carried out as described previously for *S. suis* strains and other pathogens (Velikova et al., 2016). Worms weighed between 0.55 and 0.65 g at the time of inoculation. SS2 strains were cultured in glucose and glycogen to the mid-log phase. Bacteria was collected and washed, and the concentration was adjusted to 2.5 × 10^7^ CFU (high dose) with normal saline. Ten-times diluted bacterial solution in normal saline was used as low-dose inoculum (2.5 × 10^6^ CFU). A heat-killed bacteria inoculum incubated at 100°C for 15 min was used as negative control in the high-dose and low-dose groups. Ten worms per group were each injected with 20 μL of inoculum into the lower left proleg using an insulin syringe. Groups were incubated at 37°C in sterile plastic culture plates (9 cm) without food for up to 6 d. Survival rate of worms was recorded at 1 d interval.

### Statistical analysis

Drawing of curves and histograms and statistical analysis were performed on GraphPad prism 8.0 software (San Diego, USA). Data were compared and analyzed using two-tailed or unpaired t-tests by using GraphPad prism 8.0 software. Survival curves were compared with the log-rank (Mantel-Cox) test. *P* < 0.05 was considered to be statistically significant. Unless otherwise specified, all the experiments were performed in triplicate.

## Results

### Pleiotropic effects of glycogen utilization on *S. suis* genome transcription

To investigate the transcriptional response of *S. suis* genome to exogenous glycogen, we compared the transcriptomic profiles from log phase cultures of SC19 grown in glucose and glycogen. A total of 908 DEGs were identified in glycogen-grown cultures using a > 2.0-fold change cut off compared with those in glucose-grown cultures. These DEGs account for 46.07% of the whole genome, of which 501 genes were upregulated and 407 genes were downregulated (**Table S2**).

Six upregulated and six downregulated genes with different transcription levels were randomly chosen from RNA sequencing data for qRT-PCR analysis (**Table 2**). The correlation between RNA sequencing and qRT-PCR was high (R^2^ = 0.932; **Figure S1**), which confirmed the effectiveness of the transcriptomic data in this study.

**Table 2:** Validation of RNA-Seq results by real-time quantitative PCR (qRT-PCR).

Gene locus tag	Gene	Description	Fold change of RNA-Seq	Fold change of qRT-PCR
SSUSC84_0015	*ftsH*	ATP-dependent zinc metalloprotease	-3.45	-4.76 ± 1.03
SSUSC84_0092	*rpmJ*	50S ribosomal protein L36	-7.14	-12.50 ± 1.40
SSUSC84_0249	*adhP*	Alcohol dehydrogenase	76.28	61.15 ± 3.51
SSUSC84_0270	*dnaJ*	Molecular chaperone	7.58	2.84 ± 0.65
SSUSC84_0376	*cysK*	Cysteine synthase A	-6.25	-5.88 ± 1.12
SSUSC84_0671	*mrp*	MucBP domain-containing protein	-9.09	-25.06 ± 1.01
SSUSC84_0917	*glgD*	Glucose-1-phosphate adenylyltransferase subunit	25.54	23.47 ± 2.86
SSUSC84_1079	*citZ*	Citrate synthase	-16.67	-33.33 ± 1.82
SSUSC84_1262	–	PTS transporter subunit EIIC	16.99	17.15 ± 1.25
SSUSC84_1724	*msmK*	ABC transporter ATP-binding protein	5.81	2.98 ± 0.59
SSUSC84_1871	*apuA*	Surface-anchored amylopullulanase	65.44	43.57 ± 2.07
SSUSC84_1959	*mnmG*	tRNA uridine-5-carboxymethylaminomethyl synthesis enzyme	-5.26	-6.25 ± 1.04

**Figure 1:**
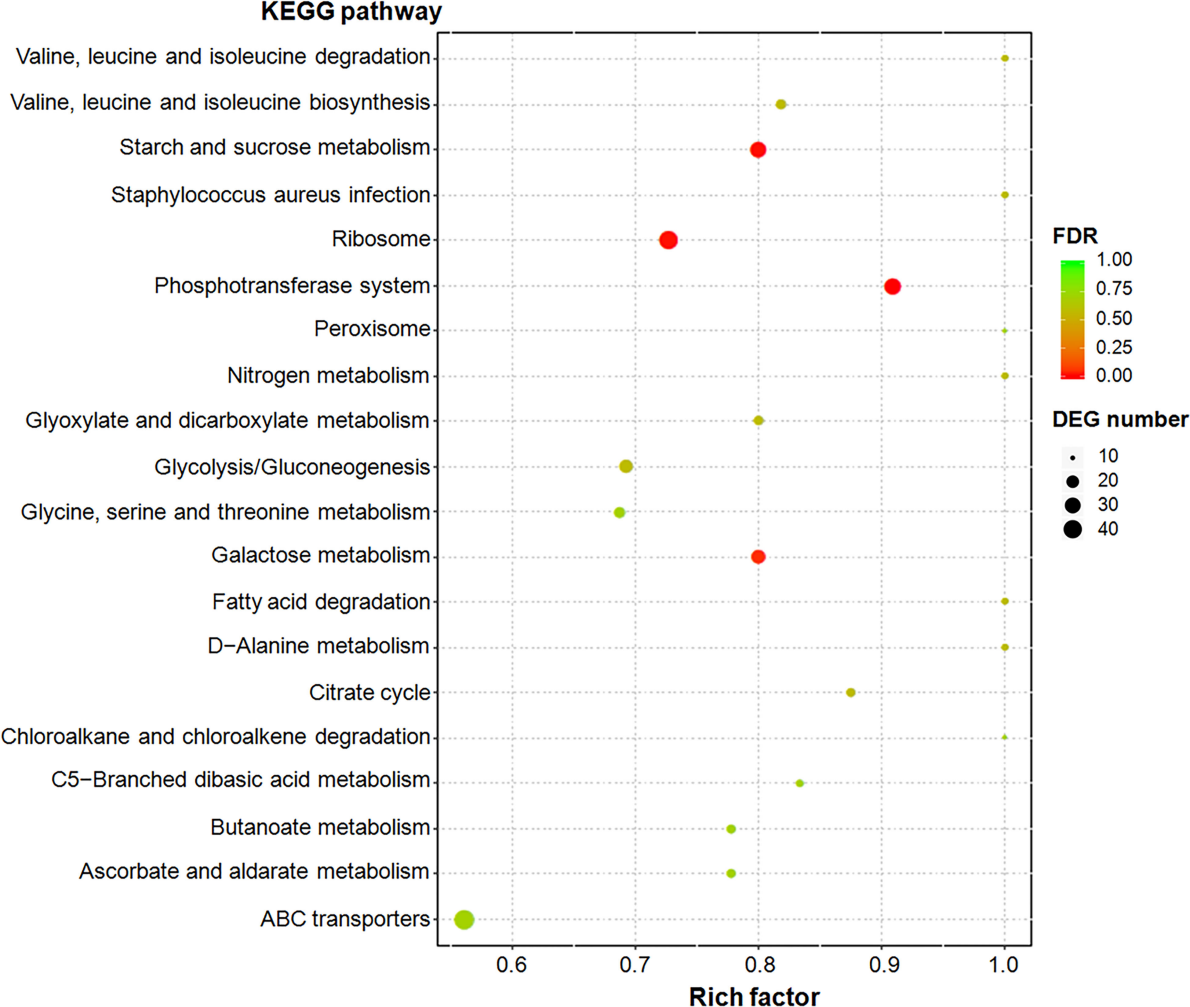
Top 20 enriched KEGG pathways of differentially expressed genes (DEGs). Rich factor refers to the ratio of the number of DEGs enriched in the pathway to the number of all genes annotated in the pathway. False discovery rate (FDR) is the corrected *P*-value value used in the multiple hypothesis testing (Zhang et al., 2014).

Functional enrichment analysis was performed on the basis of KEGG database, and DEGs were enriched in 131 KEGG pathways (**Table S3**). Among them, eight metabolic pathways, including phosphotransferase system (PTS), starch and sucrose metabolism, ribosome, galactose metabolism, valine, leucine and isoleucine biosynthesis, glycolysis/gluconeogenesis, citrate cycle (TCA cycle), and glyoxylate and dicarboxylate metabolism (Fisher’ exact test; *P* < 0.05; **Figure 1**), were significantly enriched.

Metabolic pathways with the maximum number of DEGs mainly included ATP-binding cassette (ABC) transporters, ribosome, PTS, starch and sucrose metabolism, and purine metabolism (**Figure S2**). Metabolic pathways with the maximum upregulated expression proportion of DEGs were galactose metabolism (95.00%, 19/20), PTS (93.33%, 28/30), amino sugar and nucleotide sugar metabolism (86.36%, 19/22), and starch and sucrose metabolism (82.14%, 9/11). All 46 DEGs belonging to ribosomal proteins were downregulated (100%), followed by two-component system (90%, 9/10), cysteine and methionine metabolism (81.82%, 9/11), and aminoacyl-tRNA biosynthesis (76.92%, 10/13). Moreover, genes involved in translation regulatory factors, such as translation initiation factors IF-1 and IF-3 and the translation elongation factor EF-G, were downregulated under glycogen induction in SC19.

### Effects on the carbohydrate transport system

**Figure 2:**
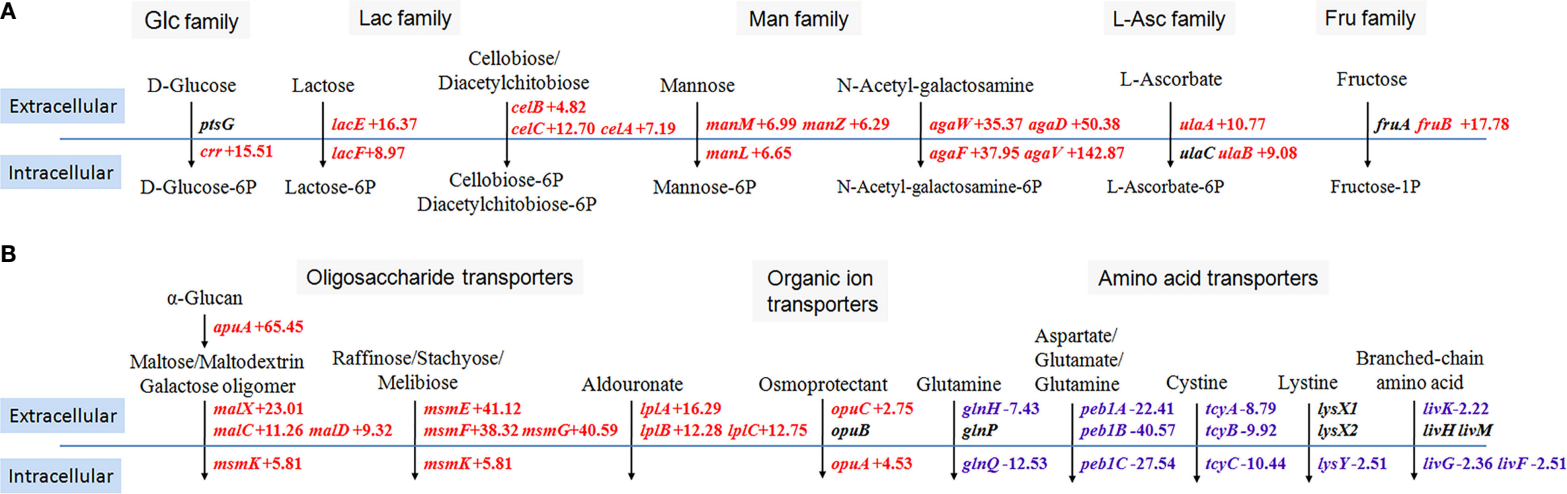
Schematic summary of differentially expressed genes involved in the phosphotransferase system **(A)** and ATP-binding cassette transporters **(B)**. Glc, glucose; Lac, lactose; Man, mannose; L-Ascorbate; Fru, fructose. Transcriptome data from bacteria grown in glucose served as the reference data. The solid blue lines indicate the cell membrane of *S. suis*. The black arrows indicate specific transport processes. Transport of carbohydrates across the cytoplasmic membrane by PTS couples with their simultaneous phosphorylation. Red color, up-regulated genes; blue color, down-regulated genes.

Exogenous glycogen can be depolymerized through cell wall-attached hydrolases in streptococci and then generated glucose, maltose, maltotriose, and maltodextrins. Glycogen degradation products were dependent on PTS and ABC transporters for transmembrane transport (Abbott et al., 2010). Therefore, we specifically summarized DEGs involved in five kinds of PTS families (**Figure 2A**) and three kinds of ABC transporters (**Figure 2B**) when comparing the transcriptome data of SC19 cultured in two carbon sources, including seven upregulated PTS, four upregulated ABC transporters, and five downregulated ABC transporters.

*crr* encodes not only the A domain of enzyme II for the PTS of glucose transport but also for the PTS of maltose, β-glucoside, α-glucoside, trehalose, and N-acetyl-muramic acid transport, and the transcription level of *crr* was significantly increased (**Figure 2A**). Other components of *crr*-cooperated PTS, including BC domain of enzyme II (PtsG), histidine protein (PtsH, also called HPr), enzyme I (PtsI), and HPr kinase/phosphorylase (HprK), showed no significant difference in transcription levels. Six other PTS, including lactose, cellobiose/diacetylchitobiose, mannose, N-acetyl-galactosamine, ascorbate, and fructose transporters, were upregulated in varying degrees. The enzyme involved in glycogen degradation (ApuA) and the ABC transporter MalXCD that transports maltose/maltodextrin and galactose oligomer were predictably upregulated. However, five amino acid transporters, such as the glutamine, cystine, and lystine transporters, were downregulated (**Figure 2B**).

### Carbohydrate metabolism analysis

**Figure 3:**
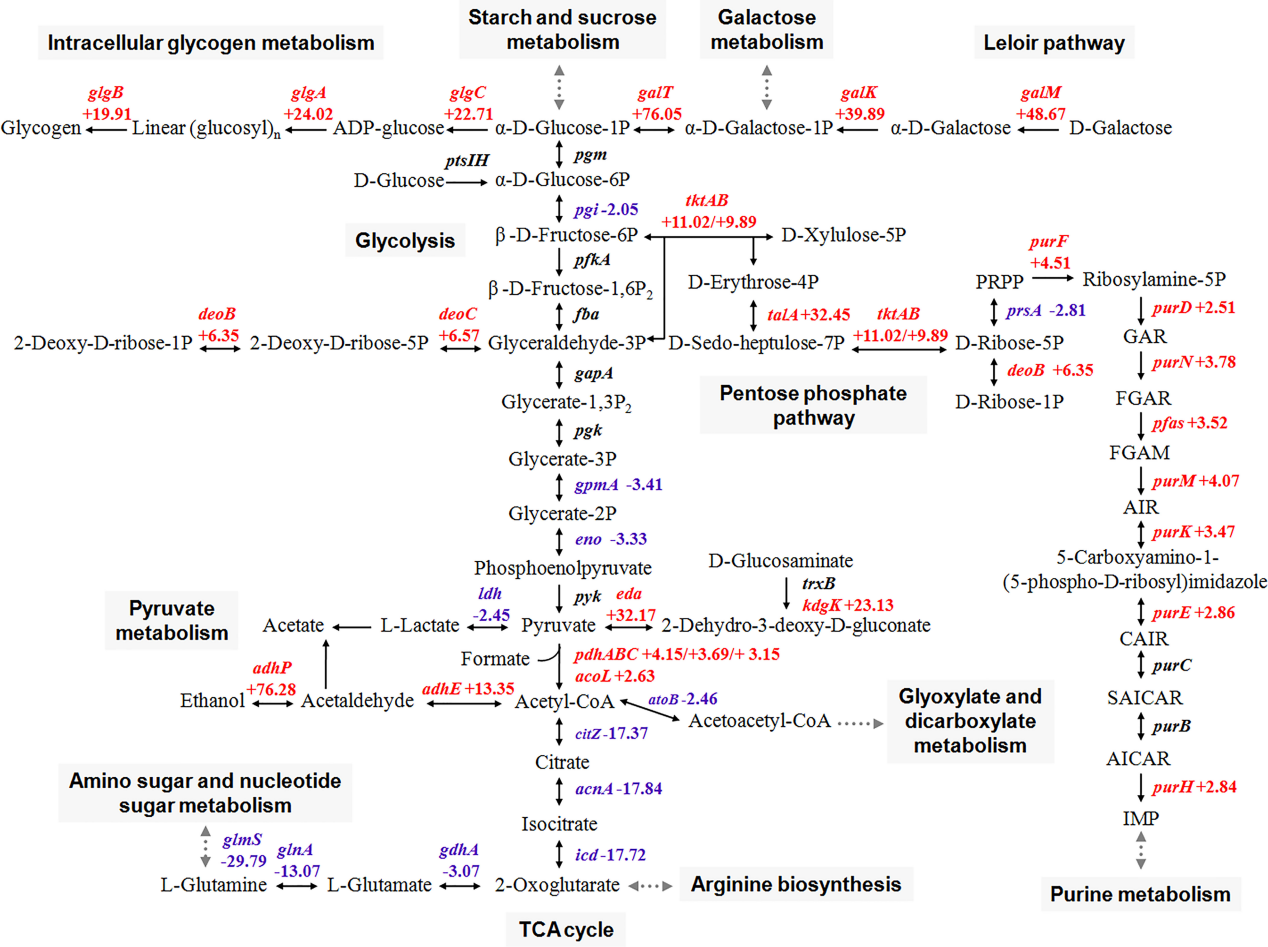
Schematic representation of main carbon metabolism pathways in *S. suis* that are differentially regulated in the presence of glycogen. Red color, upregulated genes; blue color, downregulated genes.

Although the difference in the transcriptional level of rate-limiting enzymes was insignificant, the expression of three genes of the glycolysis pathway (*pgi*, *gpmA*, and *eno*) and three genes of the fragmentary TCA cycle (*citZ*, *acnA*, and *icd*) significantly decreased (**Figure 3**). This finding suggested that the central metabolic pathway is disturbed during growth in glycogen compared with that in glucose. By contrast, pentose phosphate pathway (PPP), where fructose 6-phosphate and glyceraldehyde-3-phosphate are located, became active. Expression profiles indicated a limited flow of glucose through the main glycolysis pathway and the metabolic shift toward the PPP of *S. suis* in the presence of glycogen.

*S. suis* is a strictly facultative anaerobic bacterium that mainly metabolizes carbohydrates *via* homolactic fermentation, which reduces pyruvate into lactate (Ferrando et al., 2014). Here, analysis of the metabolic network showed that additional acetyl-CoA was formed by the reactions of oxidation and decarboxylation from pyruvate and preferentially converted into ethanol by the bifunctional acetaldehyde-CoA/alcohol dehydrogenase (AdhE) and the alcohol dehydrogenase (AdhP) (**Figure 3**). These findings indicated that growth in glycogen induces the heterofermentative growth in *S. suis*, and then leads to mixed-acid fermentation (formate, acetate, and ethanol) instead of homolactic fermentation.

The increased expression of genes associated with the Leloir pathway (*galK*, *galT*, and *galM*) which are involved in the formation of glucose-1-phosphate from galactose was observed. Glucose-1-phosphate is then preferentially promoted to glycogen synthesis *via glgABC* for energy reserves. Furthermore, the increased expression of enzymes involved in the purine metabolism was also observed.

### CcpA-mediated regulation of carbon metabolism

The CcpA regulon regulates the central carbon metabolism of *S. suis* mainly by binding either to a pseudo-palindromic *cre* motif or a novel *cre2* motif within the regulatory elements of genes, including CcpA, which was controlled by itself in a negative feedback manner (Willenborg et al., 2014). The transcriptional behavior of 77 genes and operons directly controlled by the CcpA regulon are listed in **Table S4**. The results showed that no significant difference is observed in the transcription level of 25 genes/operons. The remaining 52 genes/operons showed different degrees of transcriptional response. Among them, 34 genes/operons, such as genes involved in lactose PTS (*lacEF*), maltodextrin metabolism (*apuA* and *malXCD*), pyruvate dehydrogenase complex (*pdhABC*), intracellular glycogen synthesis (*glgCAB*), Leloir pathway (*galKT*), and *ccpA*, were upregulated. By contrast, the expression of 18 genes responsible for glycolysis (*eno*), homolactic fermentation (*ldh*), and incomplete TCA cycle (*acnA*) were downregulated. Other carbon metabolism-related genes directly regulated by CcpA, such as maltodextrin phosphorylase (*glgP*), showed no response to glycogen induction.

### Virulence-associated genes

Previous studies proposed close links between carbohydrate metabolism and virulence gene regulation in SS2 (Ferrando et al., 2014; Willenborg et al., 2014). More than 60 virulence-associated factors, such as toxins (Sly), functional enzymes (LDH, GAPDH, FBA, PGK, etc.), cell surface-associated proteins (Ssa, HtpsC, Cbp40, etc.) and regulatory factors, were identified in SS2 (Li et al., 2015; Segura et al., 2016). We analyzed the transcriptome data of 62 virulence-associated genes of SS2. Thirty-two DEGs, including 20 upregulated genes and 12 downregulated genes, were obtained (**Table 3**). Among them, seven known virulence-associated factors were highly upregulated (expression ration > 20), including haemolysin (*sly*), four adhesins (*ssa*, *abpB*, *apuA*, *gtfA*), subtilisin protease (*pepD*), and hyaluronidase (*hepI*/*III*). Three core genes (*arcABC*) involved in the arginine deiminase system (ADS) and acid resistance were also significantly upregulated. The 30 remaining genes that encode bacterial virulence factors, such as CPS biosynthesis genes (*cps*), sialic acid synthesis genes (*neu*), adhesin (*dppIV*, *atl*, *fba*, *gdpP*, etc.), and virulence marker gene *ef*, were stably expressed.

**Table 3:** Confirmed and putative bacterial virulence factors differentially expressed in glycogen compared with that in glucose.

Protein	Description	Virulence *^a^*	Fold change	*p*-value
Sly	Suilysin	Hemolysin	41.61	0
Ssa	Fibronectin-binding protein	Adhesion ECM	21.22	0
HP0197	Hypothetical protein	Adhesion ECM	6.35	2.18E-89
PdhA	Pyruvate dehydrogenase, alpha subunit	Adhesion ECM	4.15	6.54E-76
LDH	Lactate dehydrogenase	Adhesion ECM	-2.44	2.80E-50
HtpsC	Type II histidine triad protein	Adhesion ECM	-4.55	3.16E-84
PGM	Phosphoglycerate mutase	Adhesion ECM	-3.03	1.95E-75
AbpB	Amylase-binding protein B	Adhesion epithelium	120.40	0
DnaK	DnaK operon	Adhesion epithelium	7.09	2.56E-92
DnaJ	DnaK operon	Adhesion epithelium	7.58	5.18E-75
SsnA	Surface-anchored DNA nuclease	Adhesion epithelium	4.15	1.49E-115
SadP	Hypothetical protein	Adhesion epithelium	2.05	4.96E-33
ApuA	Amylopullulanase	Adhesion epithelium	65.44	0
GtfA	Glycosidase	Adhesion epithelium	85.19	3.91E-133
MRP	Muramidase released protein	Adhesion epithelium	-9.09	3.11E-263
DivIVA	Cell division initiation protein	Adhesion epithelium	-2.08	6.49E-35
GlnA	Glutamine synthetase	Adhesion epithelium	-12.5	0
Eno	Enolase	Adhesion epithelium	-3.33	9.28E-76
SrtA	Sortase A	Adhesion epithelium	-2.13	1.59E-29
GlnA	Glutamine synthetase	Adhesion epithelium	-12.5	0
PepD	Amynoacyl histidine peptidase	Subtilisin protease	120.40	0
ArcA	Arginine deaminase	Biological fitness	45.57	0
ArcB	Ornithine carbamoyltransferase	Biological fitness	68.03	0
ArcC	Carbamate kinase	Biological fitness	378.49	0
ArgR	Arginine repressor	Biological fitness	3.21	3.69E-10
HepI/III	Oligohyaluronate lyase	Hyaluronidase	24.34	2.00E-212
STP	Serine/threonine protein phosphatase	Regulatory factor	2.12	4.72E-35
STK	Serine/threonine protein kinase	Regulatory factor	2.13	4.54E-37
CcpA	Catabolite control protein A	Regulatory factor	3.60	8.26E-69
GidA	Glucose-inhibited division protein	Regulatory factor	-5.26	1.54E-130
RelA	GTP pyrophosphokinase	Regulatory factor	-3.33	2.19E-59
DltA	D-alanine-poly ligase	LTA D-alanylation	-2.70	4.62E-53

**^a^** ECM, extracellular matrix; LTA, lipoteichoic acid.

### Construction and confirmation of the mutant strain Δ*apuA*

**Figure 4:**
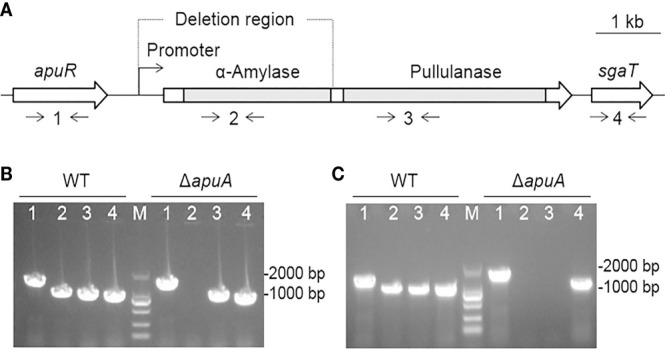
Construction and confirmation of the deletion mutant strain Δ*apuA*. **(A)** Genetic map of the loci encoding ApuA in the *S. suis* genome. Two gray bars represent the coding regions of α-amylase and pullulanase of *apuA*, respectively. Numbers 1–4 represent the detection primer pairs apuR-F/apuR-R, amy-F/amy-R, pul-F/pul-R, and sgaT-F/sgaT-R, respectively. **(B)** Combined PCR analyses of Δ*apuA*. Numbers 1–4 represent the specific DNA fragments amplified with corresponding primers in **Figure 2A**. M represents the DNA marker (100–2000 bp). **(C)** Confirmation of the transcriptional inactivation of *apuA* by real-time PCR. Numbers 1-4 represent the specific DNA fragments amplified with corresponding primers in **Figure 2A**. M represents the DNA marker.

The *apuA* gene of SC19 (6285 bp) encodes the amylopullulanase ApuA (2094 amino acids), which contains distinct α-amylase and pullulanase domains (**Figure 4A**). Located upstream of the *apuA* locus is *apuR*, a LacI family DNA-binding transcriptional regulator. Downstream of the *apuA* locus is *sgaT*, a putative ascorbate-specific PTS permease. *apuA* was deleted *via* a double-crossover method through homologous recombination to generate the Δ*apuA* strain. The definite deletion of *apuA* was confirmed through PCR (**Figure 4B**), DNA sequencing, and RT-PCR (**Figure 4C**) analyses. The RT-PCR analysis also proved that transcription activities of *apuR* and *sgaT* remain unaffected by the *apuA* deletion, which could exclude associated polarity effects.

### Growth characteristics of the WT and Δ*apuA* strains

**Figure 5:**
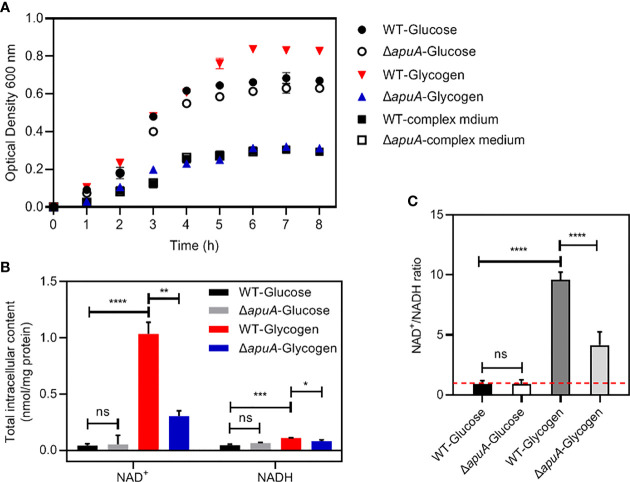
Different characteristics of WT and Δ*apuA* treated with different media. **(A)** Growth curves. Strains were grown in different media at 37°C. The turbidity of cultures was measured at 1 h intervals. **(B)** The total intracellular content of NAD^+^ and NADH. Data was presented as mean ± SEM. **(C)** The NAD^+^/NADH ratio. The total intracellular NAD^+^/NADH ratio was presented as mean ± SEM. The red dotted line in the histogram indicates the NAD^+^/NADH ratio of 1. Statistical significance was determined using unpaired Student’s *t* tests (ns, *P* > 0.05; *, *P* < 0.05; **, *P* < 0.01; ***, *P* < 0.001; ****, *P* < 0.0001).

WT and Δ*apuA* strains were analyzed for their ability to grow on glycogen and glucose as major carbon sources (**Figure 5**). Previous studies demonstrated that *S. suis* only grows to high density in complex medium in the presence of exogenous carbohydrates (Ferrando et al., 2010). Both strains grew to a low density during the entire culture period in the present work. Supplementation with glucose supported the growth of WT and Δ*apuA* strains to an OD_600_ of over 0.6. The supplementation of glycogen even supported the growth of WT to an OD_600_ of over 0.8. However, the growth of Δ*apuA* in glycogen can only reach the same optical density as that in complex medium alone, and the OD_600_ value of Δ*apuA* was significantly lower than that of WT. The results showed that the mutant loses the ability to degrade and utilize glycogen.

### Increased production of NAD^+^ and NADH

The fragmentary TCA cycle allows the synthesis of additional NADH, which is used not only for the homolactic fermentation pyruvate to lactate but also contributes to a balanced NAD^+^/NADH ratio in *S. suis* (Willenborg et al., 2015). We accordingly measured the total intracellular content of NAD^+^ and NADH (**Figure 5B**) and the NAD^+^/NADH ratio (**Figure 5C**) of the WT and Δ*apuA* strains in the presence of glucose and glycogen. The results showed that the WT strain treated with glycogen exhibited a significantly increased content of NAD^+^ and the NAD^+^/NADH ratio compared with that treated with glucose. The content of NAD^+^, NADH, and the NAD^+^/NADH ratio of *S. suis* in the presence of glucose remained unaffected by the absence of *apuA*.

### Invariant morphology and capsule characterization between WT and mutant strains

**Figure 6:**
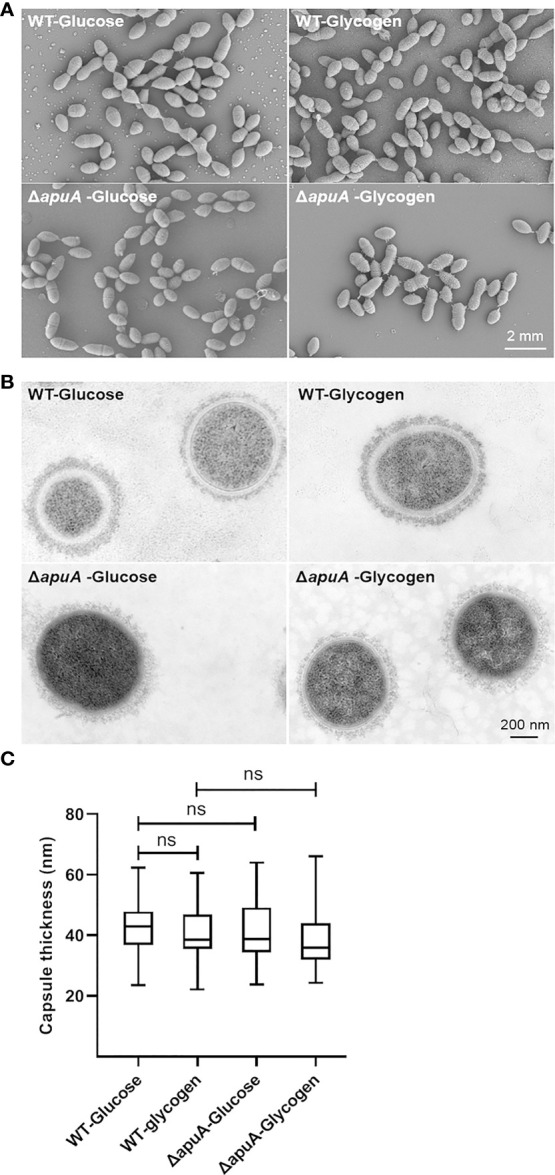
Bacterial morphology characterization of WT and Δ*apuA* in the presence of different media. **(A)** Scanning electron micrographs. **(B)** Transmission electron micrographs. **(C)** Comparative analysis of capsule thickness. Data are presented as box-and-whisker plot (minimum to maximum). Statistical analyses were performed *via* unpaired Student’ s *t* tests (ns, P > 0.05).

Transcription levels of important cell division components and cell shape determinants in *S. suis*, such as *ftsZ*, *ftsA*, *gpsB*, *ezrA*, *sepF*, *mapZ*, and peptidoglycan synthesis genes, showed no significant changes (**Table S2**). SEM and TEM analyses were accordingly performed to examine the morphology (cell length, cell width, and cell wall thickness) of WT and Δ*apuA* strains cultured in glycogen and glucose as well as investigate actual effects of glycogen utilization and *apuA* on bacterial morphology. Electron micrographs of SEM (**Figure 6A**) and TEM (**Figure 6B**) exhibited no difference between WT and Δ*apuA* grown in either glycogen or glucose.

Moreover, transcriptome data demonstrated that the expression of genes that code for the main transferases (Cps2E and Cps2F), filppase (Cps2O), polymerase (Cps2I), and translocases (Cps2B, Cps2C, and other) involved in the construction of class I CPS and four core enzymes (NeuA–D) involved in the construction of class II CPS (N-acetylneuraminic acid, namely, sialic acid) is not significantly affected by the glycogen treatment (**Table S2**). Capsule thickness of bacterial cells was measured, and the results were compared from the TEM images (**Figure 6C**). These findings confirmed the transcriptome data and showed that the bacterial morphology and capsule synthesis of *S. suis* remained unaffected by glycogen utilization and *apuA* deletion.

### Effect on hemolytic activity

**Figure 7:**
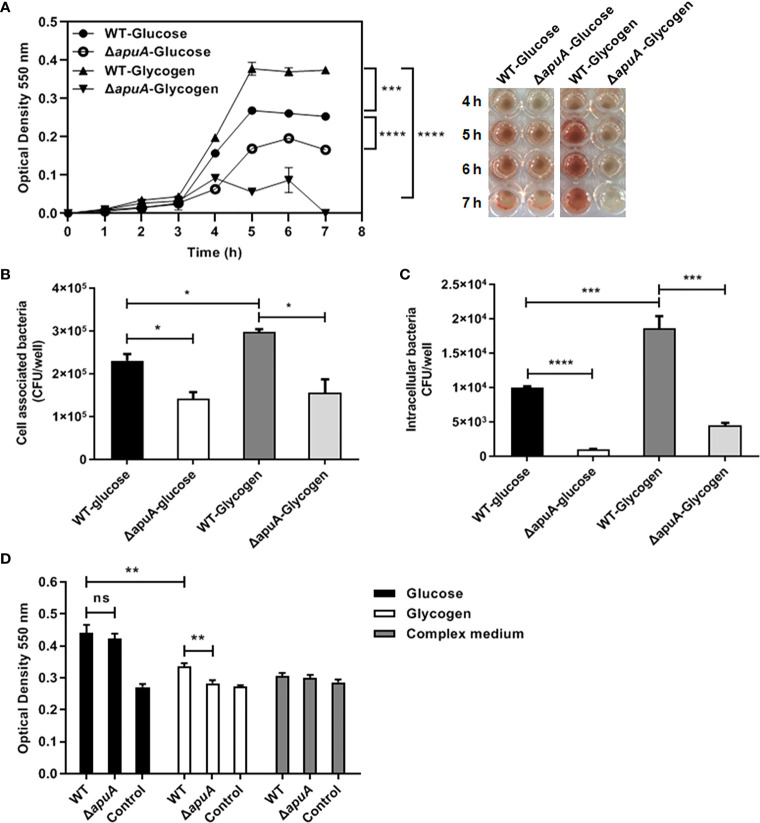
Pathogenic assays of WT and Δ*apuA* in the presence of different medium. **(A)** Hemolysis assay. Hemolysis production was quantified by analyzing the supernatants collected from WT and Δ*apuA* supplemented with glucose and glycogen. The absorbance at each hour at stable period (5, 6, and 7 h) was recorded and analyzed by pairwise comparison. **(B)** Cell-associated bacteria recovered after incubation with NPTr cells. **(C)** Pathogen invasion of NPTr cells. Extracellular bacteria were eradicated through antibiotic treatment. **(D)** Biofilm formation assay. The wells with a sterile medium and CM served as negative controls. Data points or columns are presented as mean ± SEM from three independent experiments performed in triplicate. Statistical significance was determined using the two-tailed t-test (ns, *P* > 0.05; *, *P* < 0.05; **, *P* < 0.01; ***, *P* < 0.001; ****, *P* < 0.0001).

The expression of *sly* was significantly induced (+41.61-fold) in bacteria supplemented with glycogen compared with that with glucose (**Table 3**). We measured the hourly erythrocyte hemolytic activity of culture supernatants of WT and Δ*apuA* strains in the presence of glucose and glycogen. As shown in **Figure 7A**, bacterial hemolytic activities began to increase after 4 h of incubation and then stabilized when strains were at the stationary phase. Hemolytic activities of WT in the presence of glycogen were significantly higher than those of glucose during the stable period, thereby suggesting that glycogen utilization promotes the expression of hemolysin in *S. suis*. Furthermore, the deletion of *apuA* significantly reduced the hemolytic activity of *S. suis* treated with glycogen or glucose. This finding indicated that the lack of *apuA* itself also reduced the production of hemolysin.

### Alteration in adhesion and invasion capabilities to epithelial cells

Transcriptional data of 33 adhesins were summarized. The results showed 10 upregulated, 9 downregulated, and 14 stably expressed adhesins (**Table 3**). Thus, we investigated whether the bacterial adhesion and invasion abilities of the WT strain grown in glycogen promote or inhibit the ability to adhere and invade NPTr cells. The analysis of the Δ*apuA* strain presented that the binding and/or invasion numbers of bacteria from glycogen cultures are significantly higher than those of bacteria from glucose cultures (**Figures 4B, C**). The absence of *apuA* impaired the capacity of SS2 to adhere to and invade epithelial cells in the presence of glycogen as well as glucose. Hence, glycogen utilization and ApuA contributed in promoting SS2 adhesion and invasion to host cells.

### Impaired biofilm formation

*S. suis* formed less biofilm in the presence of glycogen than that in the presence of glucose after incubation for 24 h. The absence of *apuA* led to almost no biofilm formed (**Figure 4D**). The *apuA* mutant in the presence of glucose did not significantly differ from the WT strain in the biofilm formation assay.

### Virulence performance against worms

**Figure 8:**
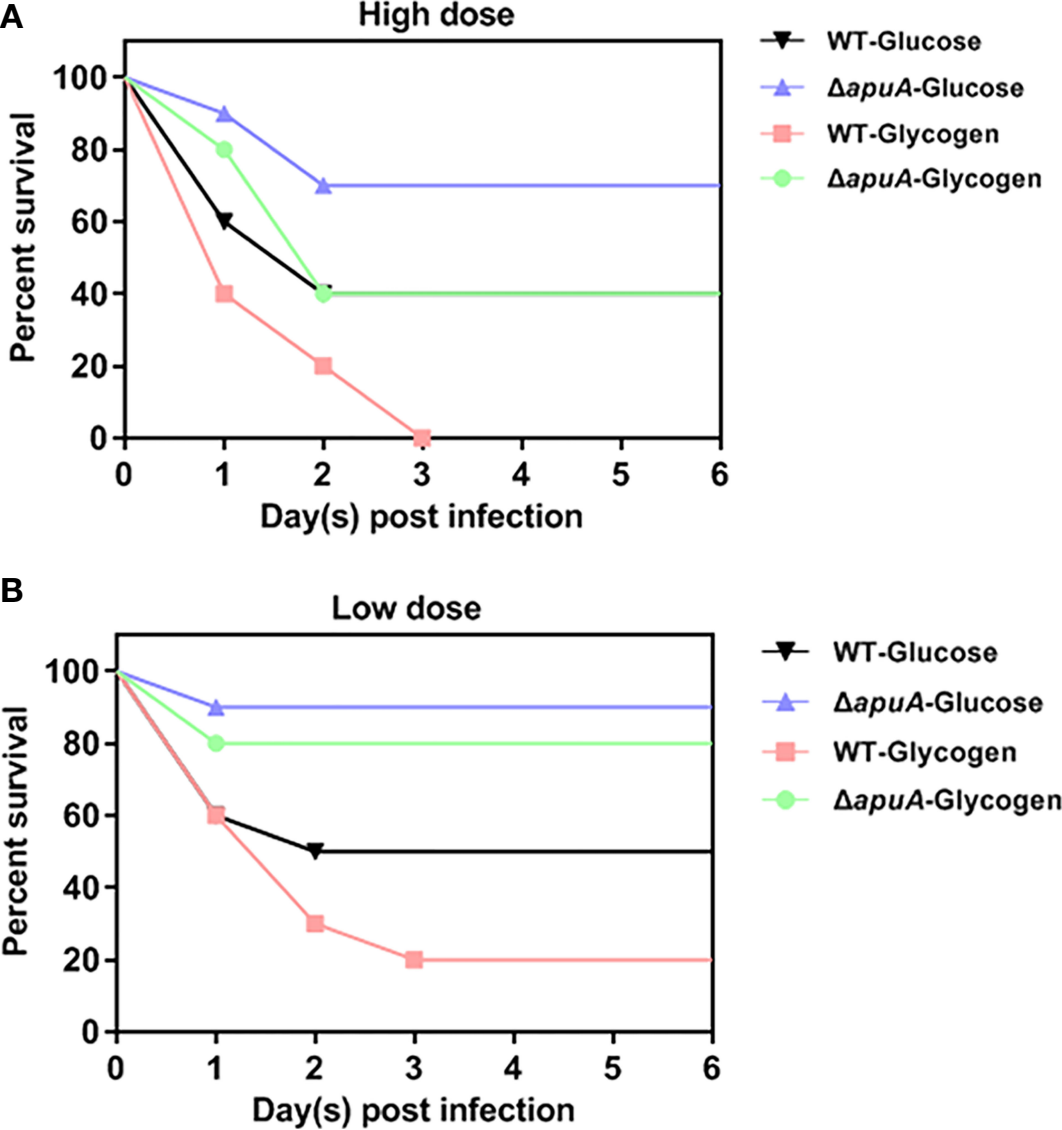
Survival curves of worms challenged with **(A)** high- and **(B)** low-dose bacteria. Ten worms were used in each group. Eight groups were inoculated with high- and low-dose inoculum. Two groups inoculated with inactivated and glycogen-induced WT bacteria were served as negative controls in the high- and low-dose groups. Survival rates of two negative control groups were 100% (not shown).

Wax worms were inoculated in parallel at high and low doses to evaluate the effect of glycogen utilization on *S. suis* infection. Survival curves for high and low doses were obtained and compared with the log-rank test after six days of observation. Livability of the WT strain in the presence of glucose was significantly higher than that in the presence of glycogen in the high dose group (**Figure 8A**). The livability of the Δ*apuA* strain grown in glucose was significantly higher than that of the WT strain grown in glucose in the low dose group (**Figure 8B**). The livability of the Δ*apuA* strain grown in glycogen was significantly higher than that of the WT strain grown in glycogen both in the high and low dose groups. Thus, glycogen treatment promoted the virulence of SS2, and ApuA can be an important virulence factor of SS2.

## Discussion

In this study, we found that glycogen could support the efficient growth of *S. suis in vitro*. We further investigated the response of the *S. suis* genome to exogenous glycogen induction through comparative transcriptome analysis. Transcriptome data showed that glycogen utilization results in transcriptional level changes of nearly 46% of the genes in the *S. suis* genome. The functional enrichment analysis of DEGs revealed that exogenous glycogen positively or negatively affects not only carbohydrate metabolism pathways but also a wide range of basic metabolic processes and stress survival pathways, such as translation, nucleotide metabolism, amino acid metabolism, lipid metabolism, and signal transduction (**Table S3**), thereby indicating the pleiotropic effects of exogenous glycogen utilization on *S. suis* transcriptome.

The glycogen treatment promoted the expression of seven PTS and three oligosaccharide transporters of *S. suis* to uptake broad-spectrum carbohydrates (**Figure 2**). An increased expression of genes involved in the Leloir pathway and intracellular glycogen metabolism was also observed (**Figure 3**). The increased expression of genes involved in carbohydrate uptake and conversions indicated that low levels of glucose trigger the uptake of alternative carbohydrates and metabolic requirements constantly adjust.

Glucose is preferentially catabolized by the glycolysis pathway and to a minor extent by PPP in *S. suis*, which mainly provides intermediates needed for anabolic reactions (Willenborg et al., 2015). The metabolic shift toward the PPP and reduced amino acid production usually represent the cellular responses to increased stresses (Nakamya et al., 2021). Increased activity of the PPP will reduce the energy-consumption through the glycolysis pathway and rapidly meet the cellular demand for NADH/NADPH to combat oxidative stress (Nakamya et al., 2021). Our data showed that the central metabolism shifts from the glycolysis pathway toward the PPP in *S. suis* after glycogen induction (**Figure 3**) and presents the possible stress responses of *S. suis* under glycogen-induced conditions. Moreover, the increased ADS (**Table 3**) can catalyze the conversion of arginine to ornithine, thereby producing ammonia, carbon dioxide, and ATP, which provides energy and protection for *S. suis* during stress (Gao et al., 2020).

Glucose has been demonstrated to repress the mixed-acid fermentation profile of *S. pneumoniae* (Paixao et al., 2015). The schematic overview of the core metabolic network showed the inhibition of homolactic fermentation and the simultaneous promotion of mixed-acid fermentation in *S. suis* supplemented with glycogen compared with those of glucose cultures (**Figure 3**), indicating a switch in the fermentation manner.

Notably, the partitioning between lactate and mixed-acid products was likely regulated at the metabolic level instead of at the transcriptional level (Paixao et al., 2015). Correspondingly, the additional NADH *in vivo* synthesized by the fragmentary TCA cycle is used for homolactic fermentation of pyruvate and contributes to a balanced NAD^+^/NADH ratio that controls the shift from homolactic to mixed-acid fermentation in *Lactococcus lactis*, *S. pneumoniae*, *Streptococcus thermophilus*, and perhaps in *S. suis* (Willenborg et al., 2015; Arioli et al., 2022). We speculated that the inhibited fragmentary TCA cycle reduces the yield of NADH and leads to the imbalance of the ratio and the transformation of fermentation manner in glycogen cultures. Subsequently, the measurement of the NAD^+^/NADH ratio supported our hypothesis (**Figure 5C**). NAD^+^ is the cofactor of many key enzymes involved in energy metabolism, such as pyruvate dehydrogenases (*pdhABC*). NAD^+^ also plays an important role in the modification of nucleic acids, proteins and other macromolecules as a reaction substrate (Paixao et al., 2015). Therefore, the significantly increased level of NAD^+^ can not only support the transformation of the fermentation manner of *S. suis* in the presence of glycogen, but also protect macromolecules such as nucleic acids and proteins from stresses.

The production of nine kinds of aminoacyl-tRNA is predicted to be disturbed because of the downregulated expression of aminoacyl-tRNA synthetases (**Table S2**). Moreover, the downregulated expression of ribosomal proteins, five amino acid transporters, and translation regulatory factors indicated that protein synthesis is inhibited, likely because low levels of glucose trigger the stringent response in *S. suis*. The classic stringent response inhibits a series of metabolic processes, such as protein synthesis, when suffering from glucose starvation to adapt to nutrient starvation in *S. suis* (Zhang et al., 2016). However, *S. suis* still maintained high growth efficiency in the presence of glycogen compared with glucose despite significant metabolic differences and inhibited protein synthesis (**Figure 5**). Further global metabolite analysis will help reveal the internal relationship between glycogen utilization and regulation of carbohydrate metabolism.

CcpA in Gram-positive bacteria primarily contributes to cellular energy homeostasis by sensing the presence of preferential sugars, such as glucose, and suppressing alternative energy providing mechanisms (Willenborg et al., 2014). The lactose PTS (*lacEF*) and maltose/maltodextrin ABC transporter (*malXCD*) were directly controlled by CcpA-dependent CCR (Willenborg et al., 2014). The expression of genes responsible for carbohydrate conversions, such as glycogenic *glgCAB* operon, Leloir pathway (*galKT*), and pyruvate dehydrogenase complex (*pdhABC*) directly controlled by CcpA, was upregulated (**Figure 3**). In addition, *pgi*, the first enzyme of the glycosis negatively and directly regulated by CcpA, was predictably downregulated because of the upregulation of *ccpA*.

However, the transcriptional performance of most genes controlled by the CcpA regulon was not evidently related to two binding motifs or CCA/CCR (**Table S4**). Three enzymes (*tpiA*, *fba*, and *eno*) of the glycosis positively regulated by CcpA were not upregulated when *ccpA* was upregulated. CcpA exhibits a narrow and direct impact on the acquisition of alternative carbohydrates. Indirect regulatory effects of CcpA are considered due to their involvement in the expression of regulatory factors (Willenborg et al., 2014). Here, the expression of genes that encode four important regulators (*stk*, *stp*, *gidA*, and *relA*) except for *ccpA* was significantly affected in *S. suis* in the presence of glycogen (**Table 3**). The serine/threonine kinase (STK) and cognate phosphatase (STP), which cooperatively regulate the metabolism, protein expression, and virulence of SS2, were upregulated (Zhang et al., 2017). The tRNA modification enzyme (GidA), which contributes to the cell division and virulence of SS2, was downregulated (Gao et al., 2020). The (p)ppGpp synthetase (RelA) induced adaptive responses of SS2 when suffering from glucose starvation and was also downregulated (Zhang et al., 2016). Therefore, the current genome transcription profiles may be due to the multiple effects of these regulatory factors. Notably, the underlying molecular basis of the CcpA regulon in Firmicutes has not been sufficiently investigated. For example, novel CcpA-binding motifs were identified in *Lactobacillus plantarum* (Chen et al., 2018) and *Bacillus licheniformis* (Xiao et al., 2021). Moreover, HPr, HPrK, and glycolytic intermediates (fructose-1,6-bisphosphate and glucose-6-phosphate), except for CcpA, play key roles in CCR and CCA; and HPr(Ser-P), a cofactor for CcpA, is also competitively inhibited by HPr(His-P) (Gorke and Stulke, 2008).

Virulence-associated factors of SS2 grown in glycogen presented differential expression in this study. In addition to the upregulated transcription level of *sly*, the transcription levels of genes that encode 19 adhesins, stress-tolerance factors, and related regulatory factors increased significantly (**Table 3**). The results of pathogenicity assays indicated that the hemolytic activity, adhesion and invasion abilities to epithelial cells, and virulence of *S. suis* are significantly enhanced because of glycogen induction, thereby confirming the validity of transcriptome data. Glucose levels in inflamed tissues may reduce because of the consumption of glucose by neutrophils and macrophages after *S. suis* infection; thus, the induction of Sly expression will release the host glycogen from damaged cells that will be degraded by ApuA and support the growth of *S. suis* (Ferrando et al., 2014). The significantly induced expression of heat shock proteins DnaJ and DnaK will help this pathogen in resisting the febrile condition and adhering to host cells (Zhang et al., 2015). The induction of AcrABC expression can provide protection for *S. suis* against acid stress (Gao et al., 2020). Therefore, the virulence response of SS2 to glycogen induction likely promotes the capacity to invade deep tissues, combat stresses, gain additional nutrients, and reach high colonization rates.

However, no suitable *in vivo* model can be used to directly investigate the interaction between pathogens and host carbohydrates. Note that high transcriptional levels of *sly* and *apuA* were observed in the joint fluid, heart and brain of SS2-infected piglets (Ferrando et al., 2014). The analysis of the transcriptional spectrum of SS2 using microarray technology also confirmed that *apuA* and other genes involved in the α-glucan metabolic pathway are highly expressed in the joint fluid, heart, and brain of infected pigs (Arenas et al., 2019). The components involved in glycogen and maltodextrin utilization are highly expressed in soft tissues of mice (Graham et al., 2006). The significant upregulated expression of *apuA* and other glycogen utilization-related enzymes in host deep tissues likely indicated that glycogen can support the growth of streptococci *in vivo*.

ApuA is an important adhesion molecule that mediates the adhesion of SS2 to porcine epithelium and mucus (Ferrando et al., 2010; Doran et al., 2016). The deletion of ApuA in the present study impaired the hemolytic activity, invasive ability, biofilm formation, and lethality of SS2 cultured in glucose (**Figures 7** and **8**). Therefore, we claimed that ApuA is not only an adhesin, but also an important virulence factor for SS2.

Taken together, our results clearly demonstrated that exogenous glycogen supports not only the efficient growth of SS2 but also promotes the metabolic adaptation, virulence gene expression, and pathogenic characteristics of this pathogen. In addition, ApuA was demonstrated to be a real virulence factor, not just an adhesin. These findings help us to understand the fitness of SS2 to available carbohydrates and pathogen–host interactions in bacterial infection. Further investigations can focus on the metabolic adaptation of this important pathogen *in vivo* to provide a theoretical basis for developing new prevention and control strategies against *S. suis* infection.

## Data availability statement

The original contributions presented in the study are publicly available. This data can be found here: NCBI, PRJNA835895.

## Author contributions

M-FT and QY contributed to conception and design of the study. M-FT, JT, F-FZ, H-QL, and S-PF performed the experiments. M-FT, H-YJ, and C-CW performed the statistical analysis. Y-LR contributed to the development of the figures. M-FT wrote the first draft of the manuscript. Y-BZ and QY performed language and logic modifications. All authors contributed to manuscript revision, read, and approved the submitted version.

## Funding

This work was supported by the National Natural Science Foundation of China (Grant No. 31960713), the Jiangxi Collaborative Innovation Foundation of Modern Agricultural Scientific Research (Grant No. JXXTCX202106), and the Jiangxi Modern Agriculture Pig-Industry Technology Research System (Grant No. JXARS-01).

## Acknowledgments

We are grateful to Professor Rui Zhou from the State Key Laboratory of Agricultural Microbiology, Huazhong Agricultural University, for providing the SC19 strain. We would also like to thank Shanghai Personal Biotechnology Co.,Ltd for providing technical support.

## Conflict of interest

The authors declare that the research was conducted in the absence of any commercial or financial relationships that could be construed as a potential conflict of interest.

## Publisher’s note

All claims expressed in this article are solely those of the authors and do not necessarily represent those of their affiliated organizations, or those of the publisher, the editors and the reviewers. Any product that may be evaluated in this article, or claim that may be made by its manufacturer, is not guaranteed or endorsed by the publisher.
