# Supplementary figures and images for: Exogenous glycogen utilization effects the transcriptome and pathogenicity of Streptococcus suis serotype 2
Source: Front Cell Infect Microbiol. 2022 Nov 9;12:938286. doi: 10.3389/fcimb.2022.938286 (PMC9683343; doi:10.3389/fcimb.2022.938286)

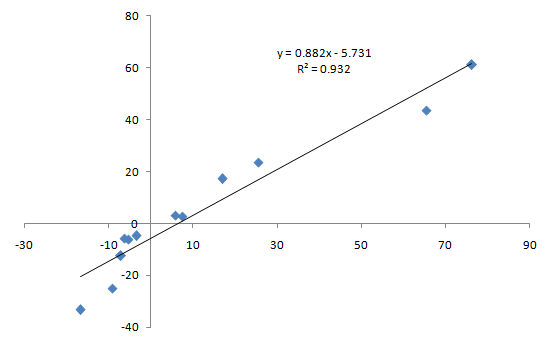

Supplement: Supplementary file 1 [file Image_1.tif]

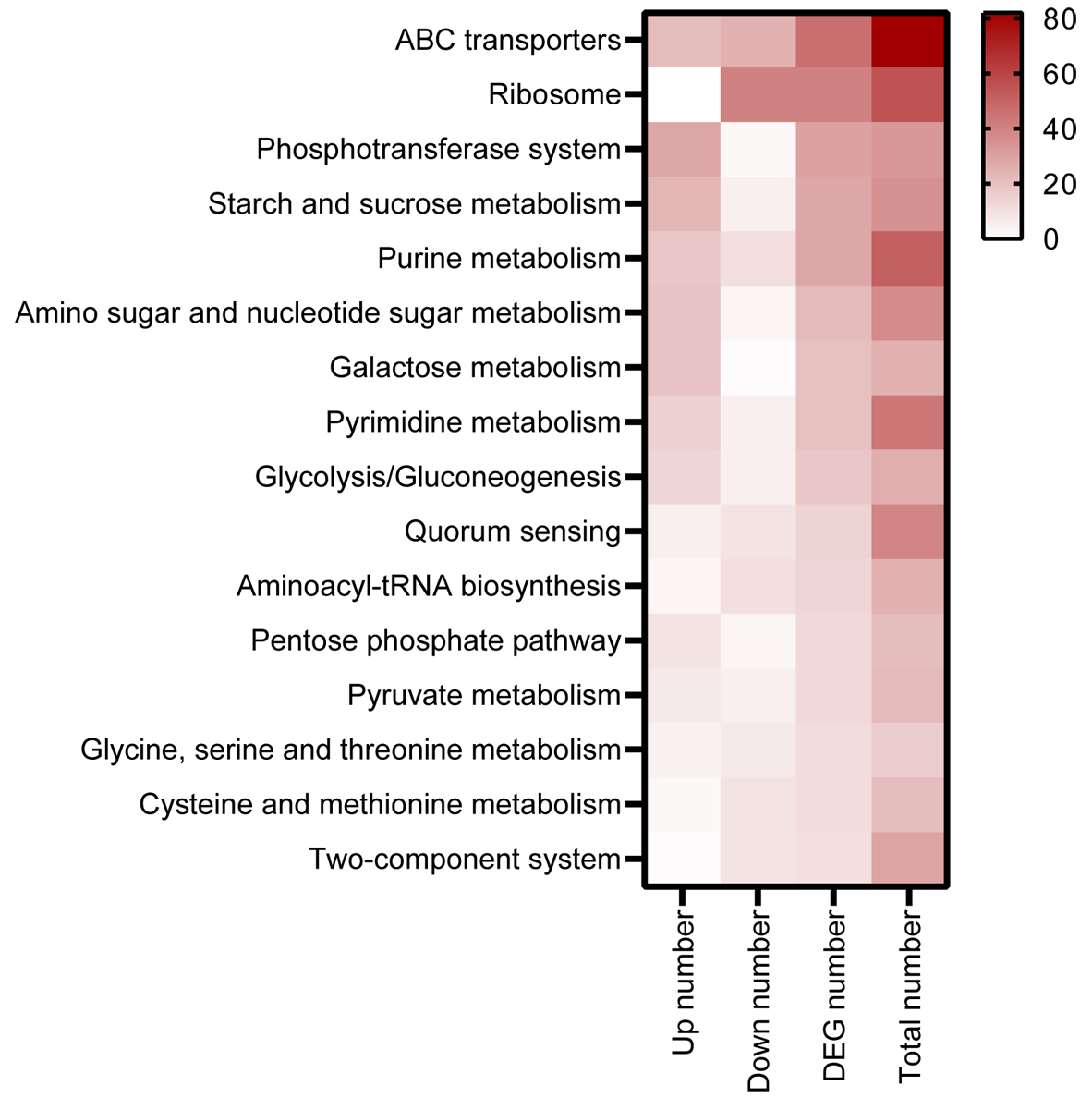

Supplement: Supplementary file 2 [file Image_2.tif]
