# Supplementary material for: Exogenous glycogen utilization effects the transcriptome and pathogenicity of Streptococcus suis serotype 2
Source: Front Cell Infect Microbiol. 2022 Nov 9;12:938286. doi: 10.3389/fcimb.2022.938286 (PMC9683343; doi:10.3389/fcimb.2022.938286)
Supplement: Supplementary file 3 [file Table_1.docx]

**TABLE S1│Primers used in this study.**

| **Primers** | **Sequence (5ʹ-3ʹ) *^a^*** | **target** |
| --- | --- | --- |
| **Real-time quantitative PCR** |  |  |
| gene291-F | CCAGCTCAAGCAAGTTCTATC | *adhP* |
| gene291-R | GCAAGGTTTCCAAGTCCA |  |
| gene1980-F | ATCGGTGGAGGTCTTGTG | *apuA* |
| gene1980-R | CTGCCTTGATACGCTCTGT |  |
| gene1352-F | AAACTCAATCCAGGTGTTCC | SSUSC84_1262 |
| gene1352-R | GTTCCCAAACTTCTTCTCCA |  |
| gene995-F | ATGGCGTGACAATCAAGAG | *glgD* |
| gene995-R | TCAAGTATGAAGGCGAAGTTT |  |
| gene312-F | CTCGTCAAGGGGATGATTT | *dnaJ* |
| gene312-R | GTTTGGCACCTGAACCTG |  |
| gene1827-F | GAGATGGTTTCAAGGTGGAG | *msmK* |
| gene1827-R | CGATGGAAGATGGATAAGTGT |  |
| gene14-F | AATGTTTGTTGGTGTCGGT | *ftsH* |
| gene14-R | GAGTTGGTTGAGGGTTTGTT |  |
| gene2073-F | GCCAAACCACTTTTCATTCT | *mnmG* |
| gene2073-R | CTGTATTGCGACCTTCTGG |  |
| gene744-F | ATCACGCTATGTTCTTGTTCC | *mrp* |
| gene744-R | CATTTGTTCCTGGTGTCGT |  |
| gene113-F | GAAAGTAAGACCATCGGTCAA | *rpmJ* |
| gene113-R | GGGCAAATCACCATAACAC |  |
| gene431-F | AAAATGGCTGGGTTCCTT | *cysK* |
| gene431-R | CTGCCGAAATACCTTGGA |  |
| gene1161-F | TGTTGTTGGCTATTCGTGAG | *citZ* |
| gene1161-R | CGCTTCATCGTCTGTTCC |  |
| 16S-F | ACTCCTACGGGAGGCAGCA | 16S rRNA |
| 16S-R | ATTACCGCGGCTGCTGG |  |
| **General PCR amplification** |  |  |
| Aup-F | CATGCATGCTCGCGTGTCATCAACCATCC | Upstream homologous fragment of *apuA* |
| Aup-R | GTCGTCGACACGTTTTCTTAAATGTTAACGGT |  |
| Adown-F | TCCCCCGGGTTGTCACAGATGCCTATACAGGA | Downstream homologous fragment of *apuA* |
| Adown-R | CGGGAATTCTGGTAAGGTCCAAGACAAGGTC |  |
| Erm-F | GCGTCGACCTTAGAAGCAAACTTAAGAGT | Erythromycin resistance gene |
| Erm-R | TACGTCGACATCGATACAAATTCCCCGTAG |  |
| apuR-F | ATGGATTTCCTTCAAACCCCA | *apuR* |
| apuR-R | CTATTCTGCTTGTGCCTCGCC |  |
| amy-F | CTGAGACACCAGGAACGATTG | Partial coding sequence of the α-amylase of ApuA |
| amy-R | TCCACACCCAATTCTTTCAAGT |  |
| pul-F | CTATTTACACCAACCCTTATTTTG | Partial coding sequence of the pullulanase of ApuA |
| pul-R | GTAAAGGCAAAGTAGCTCTGTG |  |
| sgaT-F | ATGACGACATTAGCCGATGTGG | *sgaT* |
| sgaT-R | CTACTGAGGTGTAGTCTCCCTTTCA |  |

***^a^*** Underlined sequences represent the restriction sites. All primers were designed in this study.
