# Supplementary material for: Exogenous glycogen utilization effects the transcriptome and pathogenicity of Streptococcus suis serotype 2
Source: Front Cell Infect Microbiol. 2022 Nov 9;12:938286. doi: 10.3389/fcimb.2022.938286 (PMC9683343; doi:10.3389/fcimb.2022.938286)
Supplement: Supplementary file 6 [file Table_4.docx]

**TABLE S4│Summary of the transcriptional data of specific genes directly controlled by the CcpA regulon.**

| **Locus tag *^a^*** | **Gene** | **Description** | **Control phase*^b^*** | **Regulation mode*^c^*** | **Binding motif** | **Fold change** |
| --- | --- | --- | --- | --- | --- | --- |
| SSUSC84_0182 | *pflB* | Formate acetyltransferase | exp/stat | CCA | *cre*/*cre2* | 21.91 |
| SSUSC84_0250 | *adhE* | Bifunctional acetaldehyde-CoA/alcohol dehydrogenase | exp | CCR | *cre* | 13.35 |
| SSUSC84_0641 | *glpK* | Glycerol kinase | exp | CCR | *cre* | 22.56 |
| SSUSC84_0970 | *ldh* | L-lactate dehydrogenase | exp/stat | CCA | *cre* | -2.45 |
| SSUSC84_1662 | *pdhA* | Pyruvate dehydrogenase E1 component, alpha subunit | exp | CCR | *cre*/*cre2* | 4.15 |
| SSUSC84_0010 | - | Septum formation initiator protein | NS | NS | *cre2* | -2.40 |
| SSUSC84_0218 | - | ABC transporter ATP-binding protein | NS | NS | *cre2* | 3.69 |
| SSUSC84_1227 | *peb1B* | Amino acid ABC transporter permease | stat | CCR | *cre2* | -37.71 |
| [SSUSC84_1636](https://www.kegg.jp/dbget-bin/www_bget?sss:SSUSC84_1636) | - | Aspartate kinase | NS | NS | *cre*/*cre2* | -2.13 |
| SSUSC84_1763 | *leuA* | 2-isopropylmalate synthase | NS | NS | *cre2* | -3.56 |
| SSUSC84_1706 | *ilvB* | Acetolactate synthase large subunit | stat | CCR | *cre2* | -2.16 |
| SSUSC84_0303 | *asnB* | L-asparaginase | NS | NS | *cre2* | -3.16 |
| SSUSC84_0920 | - | Extracellular amino acid-binding protein | stat | CCR | *cre2* | -3.70 |
| SSUSC84_0647 | - | Ibonucleoside-triphosphate reductase | exp/stat | CCR | *cre2* | 4.97 |
| SSUSC84_0700 | *pyrR* | PyrR bifunctional protein | stat | CCR | *cre2* | NS |
| SSUSC84_0978 | *deoA* | Pyrimidine-nucleoside phosphorylase | exp | CCR | *cre2* | 11.62 |
| SSUSC84_1780 | *purA* | Adenylosuccinate synthetase | NS | NS | *cre2* | -2.50 |
| SSUSC84_0238 | *gla* | Glycerol facilitator-aquaporin | exp/stat | CCA | *cre* | -6.06 |
| SSUSC84_1394 | *livK* | Branched-chain amino acid ABC transporter | exp | CCA | NS | -2.22 |
| SSUSC84_1385 | - | Surface-anchored 5'-nucleotidase | stat | CCA | NS | 10.45 |
| SSUSC84_0976 | *cdd* | Cytidine deaminase | exp | CCR | NS | 4.91 |
| SSUSC84_0300 | *fba* | Fuctose-bisphosphate aldolase | stat | CCA | NS | NS |
| SSUSC84_0316 | *galK* | Galactokinase | NS | CCR | *cre* | 39.89 |
| SSUSC84_0467 | *tpi* | triosephosphate isomerase | stat | CCA | NS | NS |
| SSUSC84_0611 | *pgm* | phosphomannomutase | exp | CCR | *cre* | NS |
| SSUSC84_0916 | *glgC* | Glucose-1-phosphate adenylyltransferase | exp/stat | CCR | *cre*/*cre2* | 22.71 |
| SSUSC84_0937 | *lacE* | PTS transporter subunit EIIBC | exp/stat | CCR | *cre2* | 16.37 |
| SSUSC84_1263 | - | N-acetylmannosamine-6-phosphate 2-epimerase | exp/stat | CCR | *cre* | 71.20 |
| SSUSC84_0340 | *glgP* | Glycogen phosphorylase | exp/stat | CCR | *cre* | NS |
| SSUSC84_1350 | *eno* | Enolase | exp/stat | CCA | *cre2* | -3.33 |
| SSUSC84_1398 | *dexB* | Glucan 1,6-alpha-glucosidase | stat | CCA | *cre2* | 3.64 |
| SSUSC84_1399 | *gtfA* | Sucrose phosphorylase | NS | NS | *cre* | 85.19 |
| SSUSC84_1857 | *pgi* | Glucose-6-phosphate isomerase | exp | CCR | *cre2* | -2.05 |
| SSUSC84_1871 | *apuA* | Surface-anchored amylopullulanase | NS | NS | *cre* | 65.44 |
| SSUSC84_1933 | *malX* | Extracellular solute-binding protein | exp/stat | CCR/CCA | *cre* | 23.01 |
| SSUSC84_1945 | - | Beta-glucosidase | NS | NS | *cre* | 3.50 |
| SSUSC84_1947 | - | Beta-glucosidase | exp | CCA | *cre* | NS |
| SSUSC84_0380 | *yvyD* | Sigma 54 modulation protein | exp | CCR | *cre* | 12.91 |
| SSUSC84_0466 | *tufA* | Elongation factor Tu | NS | NS | *cre* | NS |
| SSUSC84_1212 | *prfC* | Peptide chain release factor 3 | NS | NS | *cre2* | NS |
| SSUSC84_0215 | - | Transcriptional regulator | NS | NS | *cre2* | NS |
| SSUSC84_0341 | - | GntR family regulatory protein | NS | NS | *cre*/*cre2* | -2.25 |
| SSUSC84_0586 | - | conserved hypothetical protein | exp | CCR | *cre* | 2.19 |
| SSUSC84_0951 | - | Putative phage repressor-like protein | exp | CCA | *cre2* | NS |
| SSUSC84_1205 | *lplB* | Sugar ABC transporter permease | NS | NS | *cre* | 12.28 |
| SSUSC84_1235 | *ccpA* | Catabolite control protein A | stat | CCA | *cre* | 3.60 |
| SSUSC84_1848 | - | MarR family regulatory protein | NS | NS | *cre2* | NS |
| SSUSC84_1872 | - | LacI family regulatory protein | NS | NS | *cre* | 3.96 |
| SSUSC84_0500 | *cps2A* | Integral membrane regulatory protein | exp | CCA | *cre* | NS |
| SSUSC84_1689 | - | D-alanyl-D-alanine carboxypeptidase | NS | NS | *cre2* | 2.20 |
| SSUSC84_1795 | - | Cell envelope proteinase | NS | NS | *cre2* | NS |
| SSUSC84_0769 | - | Plasmid replication protein | NS | NS | *cre2* | NS |
| SSUSC84_1506 | *pflC* | Formate-lyase activating enzyme | exp | CCR | *cre* | 5.55 |
| SSUSC84_1843 | *radA* | DNA repair protein RadA | stat | CCR | NS | -3.79 |
| SSUSC84_1985 | *htrA* | Serine protease | stat | CCA | *cre2* | NS |
| SSUSC84_0993 | - | Phosphate ABC transporter | NS | NS | *cre2* | NS |
| SSUSC84_1022 | - | Cation efflux family protein | stat | CCR | *cre2* | NS |
| SSUSC84_0931 | - | ABC transporter, ATP-binding protein | stat | CCR | *cre*/*cre2* | 2.09 |
| SSUSC84_1380 | - | Phosphoesterase | NS | NS | *cre2* | NS |
| SSUSC84_1604 | - | Gamma-glutamyl hydrolase | exp | CCR | *cre2* | NS |
| SSUSC84_1782 | *ssnA* | LPXTG cell wall anchor domain-containing protein | stat | CCA | *cre* | 4.15 |
| SSUSC84_1826 | - | ssDNA-binding protein | NS | NS | *cre2* | NS |
| SSUSC84_0573 | - | Low temperature requirement A protein | NS | NS | *cre2* | NS |
| SSUSC84_0654 | *crp* | Crp/Fnr family transcriptional regulator | exp/stat | CCR | *cre* | 12.07 |
| SSUSC84_0954 | - | ABC transporter ATP-binding membrane protein | stat | CCR | *cre2* | -4.25 |
| SSUSC84_0532 | - | Hypothetical protein | stat | CCR | *cre2* | NS |
| SSUSC84_0682 | - | Putative exported protein | NS | NS | *cre2* | -5.66 |
| SSUSC84_0784 |  | Putative membrane protein | stat | CCR | *cre2* | NS |
| [SSUSC84_0933](https://www.kegg.jp/dbget-bin/www_bget?sss:SSUSC84_0933) | - | Putative membrane protein | NS | NS | *cre2* | 2.22 |
| SSUSC84_1062 | - | Putative membrane protein | NS | NS | *cre2* | NS |
| SSUSC84_1264 | *sly* | Suilysin | NS | NS | *cre2* | 41.61 |
| SSUSC84_1333 | - | Putative lipoprotein | stat | CCR | *cre2* | 9.84 |
| SSUSC84_0938 | *lacF* | PTS transporter subunit EIIA | exp | CCR | NS | 8.97 |
| SSUSC84_1705 | *ilvH* | Acetolactate synthase small subunit | stat | CCR | NS | -2.48 |
| SSUSC84_1202 | *sacC* | Beta-fructofuranosidase | exp | CCR | NS | 15.10 |
| SSUSC84_1263 | - | N-acetylmannosamine-6-phosphate 2-epimerase | exp | CCR | NS | 71.20 |
| SSUSC84_1947 | - | Beta-glucosidase | exp | CCA | NS | NS |

*^a^*Only the first gene in the regulated operon was listed in the table.

*^b^*exp, exponential phase; stat, stationary phase.

*^c^*CCA, carbon catabolite activation; CCR, carbon catabolite repression.
